# Effect of DC Plasma Electrolytic Oxidation on Surface Characteristics and Corrosion Resistance of Zirconium

**DOI:** 10.3390/ma11050723

**Published:** 2018-05-03

**Authors:** Maciej Sowa, Wojciech Simka

**Affiliations:** Faculty of Chemistry, Silesian University of Technology, B. Krzywoustego Street 6, 44-100 Gliwice, Poland

**Keywords:** zirconium, anodization, plasma electrolytic oxidation, biomaterials, corrosion, electrochemical impedance spectroscopy, potentiodynamic polarization

## Abstract

Zr is a valve metal, the biocompatibility of which is at least on par with Ti. Recently, numerous attempts of the formation of bioactive coatings on Zr by plasma electrolytic oxidation (PEO) in solutions that were based on calcium acetate and calcium β-glycerophosphate were made. In this study, the direct current (DC) PEO of commercially pure zirconium in the solutions that contained Ca(H_2_PO_2_)_2_, Ca(HCOO)_2_, and Mg(CH_3_COO)_2_ was investigated. The treatment was conducted at 75 mA/cm^2^ up to 200, 300, or 400 V. Five process stages were discerned. The treatment at higher voltages resulted in the formation of oxide layers that had Ca/P or (Mg+Ca)/P ratios that were close to that of hydroxyapatite (Ca/P = 1.67), determined by SEM/EDX. The corrosion resistance studies were performed using electrochemical impedance spectroscopy (EIS) and DC polarization methods. *R*(*Q*[*R*(*QR*)]) circuit model was used to fit the EIS data. In general, the coatings that were obtained at 200 V were the most corrosion resistant, however, they lacked the porous structure, which is typical for PEO coatings, and is sought after in the biomedical applications. The treatment at 400 V resulted in the formation of the coatings that were more corrosion resistant than those that were obtained at 300 V. This was determined mainly by the prevailing plasma regime at the given process voltage. The pitting resistance of Zr was also improved by the treatment, regardless of the applied process conditions.

## 1. Introduction

There are many requirements posed implantable medical materials, like sufficient mechanical strength, good biocompatibility, and chemical stability. Although polymeric biomaterials are gradually increasing their significance in the biomedical applications [1,2], metals and alloys are still unparalleled as dental and orthopedic implants [3]. Titanium and its alloys are among the most popular metallic biomaterials because of the excellent corrosion resistance and their very good strength-to-weight ratio. Ti-6Al-4V, in particular, is extensively used due to the presence of α+β Ti phase, thus improving its mechanical strength even further. However, the presence of V in the alloy caused concern because of its cytotoxicity [4], while Al is known to be neurotoxic and it might be responsible for Alzheimer’s disease [5]. Meanwhile, more interest has been given to β phase Ti alloys, which exhibit lower Young modulus, thereby decreasing the stress-shielding effect [6,7,8].

Zirconium is a neutral element when it comes to the phase stabilization in Ti alloys, however, it exhibits excellent biocompatibility, and it is often present in their composition [7,9,10,11,12]. Although Zr is mostly resistant to general corrosion it displays limited resistance to pitting corrosion in chloride environments [7,13]. Zirconium oxide (or zirconia) has attracted researchers’ interest as early as in the 1960s [14] and it has been successfully applied to total hip replacement heads. Yet, it was discovered that it had experienced bad in vivo aging performance and it was proposed to use femoral heads that were made of both alumina and zirconia instead [15]. Zirconia was also utilized in tandem with titania and hydroxyapatite (HA) for dental implants [16]. Furthermore, Olmedo et al. [17], through in vivo biocompatibility studies using microparticles of Ti and Zr, showed that the concentration of zirconium in human tissues was lower than that of titanium, however, the particles’ size and shape had a deciding impact on the results.

Good fixation of an implant determines its successful application and the use of bioactive biomaterials, which are capable of direct binding with bone tissue via HA layer that is formed in the organism at the implantation site, is often practiced. Bioceramics, especially calcium orthophosphates (Ca-P), were shown to display such behavior. HA is the key representative of this group [18]. There were many attempts of functionalization of this material with other elements, most notably with magnesium and strontium [19,20,21,22]. For instance, Mg^2+^ as a divalent ion can substitute calcium ions in HA, and it is an important bioelement from the point of view of bone growth [19].

The improvement of bioactivity of metals can be achieved by the application of surface modification methods, resulting in the formation of Ca-P ceramic coatings, which improve the corrosion resistance of the underlying metal at the same time [23,24]. Plasma electrolytic oxidation (PEO) is a surface treatment, which produces oxide coatings on metals in passivating electrolytes. The process can be conducted under the direct current (DC), alternating current (AC), or pulsed bipolar regimes [25,26,27,28]. The oxide layers produced by PEO are porous and relatively thick (up to several hundred microns) with the possibility to incorporate the electrolyte species into their structure. The pores in the coatings, on the one hand, decrease the corrosion resistance of the protective layer, but on the other hand, it increases the area of contact between the implant’s surface and the organism, which results in better anchoring, and consequently, better bioactivity [29].

Initially, apart from the Zr-containing Ti alloys [8,30,31], relatively small interest was devoted to the research concerning PEO of Zr-based materials. However, since 2007, many reports of PEO of Zircaloys [32,33] and other Zr alloys [34,35,36], as well as pure zirconium [37,38,39,40,41,42,43,44,45,46,47,48], were to be found. Most of the studies were mainly concerned with the surface characterization of the coatings that were obtained in the silicate-based electrolytes [34,35,36,41,44,49,50,51,52]. Among the groups that were working on the synthesis of bioactive PEO films on Zr, only a few papers can be found on the treatments that were conducted in the electrolytes other than calcium acetate + calcium β-glycerophosphate systems [41,43,46,53]. Even less consideration was dedicated to the study of corrosion of such bioactive coatings in physiological media [41,48].

The authors of the research have already conducted some studies on DC PEO of zirconium in silicate solutions [54,55]. This paper is a continuation of the two previous reports concerning PEO of niobium [56] and tantalum [57] that were carried out in the electrolytes that contained calcium hypophosphite, calcium formate, and magnesium acetate. Ha et al. [46] also treated Zr under DC conditions in the presence of Mg(CH_3_COO)_2_, however, their investigations were limited to the surface characterization of the coatings, and their subsequent chemical modification in H_2_SO_4_ and NaOH. In addition, no heed was paid to the process stages of the treatment. Therefore, the present research is devoted to elucidating the effect of the process voltage and the composition of the treatment baths on the surface properties of the obtained coatings as well as their corrosion resistance in a simulated physiological environment using electrochemical impedance spectroscopy (EIS) and polarization methods.

## 2. Materials and Methods

**Figure 1:**
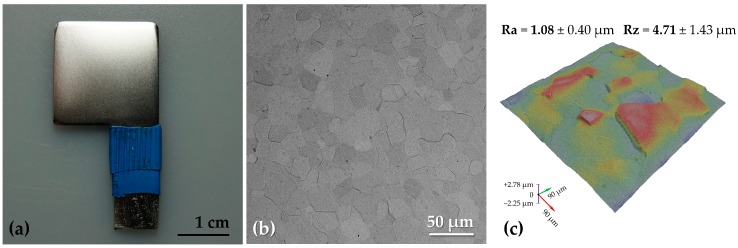
Zirconium specimen prior to plasma electrolytic oxidation (PEO) treatment viewed in macro (**a**) and micro (**b**,**c**) scales. The micrograph (**b**) and the three-dimensional (3-D) roughness mapping (**c**) were acquired using SEM.

Pure zirconium samples were obtained from a 0.6 mm metal sheet (BIMO Metals, Wrocław, Poland) by cutting it into samples resembling a letter “p” (Figure 1a). The surface of the specimens that were used in the research was limited by the use heat-shrink tubing as shown in the figure. The total surface area exposed to the treatment was 20 mm × 20 mm × 2 (the thickness of the samples was ignored). Zr surfaces were pretreated by mechanical grinding using SiC water-proof abrasive papers (grades 320, 600, and 800 used in succession), degreasing in the acetone-water mixture (1:1 vol; 5 min with ultrasounds), and chemical etching (1 M HF, 4 M H_2_SO_4_; 2 min with mild mixing). The last step, which was performed to remove any SiC particles that were remaining after the grinding step, was followed by the rinsing of the samples with copious amounts of deionized water (Milli-Q purifier, Merck Millipore, Darmstadt, Germany) and drying in a stream of warm air. The samples that underwent the pretreatment (Bare Zr samples) served as a reference in the research and were used in the final step of the treatment—plasma electrolytic oxidation.

PEO of the Zr specimens was realized in a two-step DC method, as described earlier for Nb and Ta [56,57]. The method consisted of an initial galvanostatic stage (constant current density; *i* = 75 mA/cm^2^), which was performed until one of the three limiting anodization voltages was reached (*U*_L_ = 200, 300 or 400 V). At this point of the procedure, the process was shifted to voltage control (potentiostatic) and the current density dropped with time. The treatment was realized until the total processing time amounted to 5 min. At this point, the samples were washed in tap water and then in deionized water, which was followed by drying in the stream of warm air. The second step of the method was applied to improve the tightness of the oxide coatings and to minimize the imperfections that were obtained at the peak voltage of the previous step [56,57,58]. The PEO process was performed in one of three studied aqueous electrolytes:

- Ca1P2: 0.5 M Ca(H_2_PO_2_)_2_ solution; Ca/P molar ratio = 0.5;
- Ca2P1: 0.1 M Ca(H_2_PO_2_)_2_ + 0.3 M Ca(HCOO)_2_ solution; Ca/P molar ratio = 2.0; and,
- (CaMg)2P1: 0.5 M Ca(H_2_PO_2_)_2_ + 1.5 M Mg(CH_3_COO)_2_ solution; (Ca+Mg)/P molar ratio = 2.0.

The composition of the electrolytes was chosen experimentally and it was aimed at obtaining the samples having the atomic surface concentration that resembled the Ca/P or (Ca+Mg)/P ratios characteristic of hydroxyapatite (Ca/P = 1.67). 

The surface of the samples was observed during the PEO by a digital camera (Sony Alpha ILCE-5000, Tokyo, Japan). In addition, just after the samples were prepared, their macroscopic images were taken to show the uniformity, color, and the overall quality of the obtained surfaces. All of the photographs were taken under the same lighting conditions.

The PEO oxide coatings were analyzed using a scanning electron microscope (SEM; Phenom ProX, Phenom World BV, Eindhoven, The Netherlands), which was operated either at an accelerating voltage of 10 or 15 kV with a back-scattered electrons (BSE) detector. Both planar and cross-sectional images of the coatings were taken. At least three images of each sample were used for the analyses. The SEM was equipped with an integrated energy-dispersive X-ray spectrometer (EDX), which was utilized to obtain the elemental composition of the films. Microroughness of the studied surfaces was also determined using the SEM in a roughness-mapping mode. Surface roughness maps for the analyses had 270 µm × 270 µm. From each map, four surface profiles were taken, and were used to determine the Ra and Rz roughness factors, as defined by the following formulae [59]:

(1) $$\mathrm{Ra}=\frac{1}{l}\;\overset{l}{\underset{0}{\int }}r\left(x\right)dx,$$

(2) Rz=Rp+Rv ,

where *l* is the sampling length (70–270 µm), *r*(*x*) is the roughness profile in the *x*-direction, which is an absolute deviation of the profile (in the *z*-direction) from the mean line, while Rp and Rv correspond to the greatest peak height and the deepest valley depth of the profile, respectively.

The thickness of the coatings was calculated by taking an arithmetic mean from the five thinnest and the five thickest points of the PEO films from 4–8 SEM cross-sections. To represent the maximum and minimum thicknesses of the coatings the standard deviations (SD) were calculated for each image and the greatest value for every coating corresponded to the thickness range.

The electrochemical corrosion experiments were carried out at 37 °C in naturally aerated Ringer’s solution (8.6 g/dm^3^ NaCl, 0.3 g/dm^3^ KCl and 0.48 g/dm^3^ CaCl_2_·6H_2_O; Fresenius Kabi, Warsaw, Poland). The zirconium specimens served as working electrodes, while the role of the reference and counter electrodes were filled by saturated calomel electrode (SCE) and platinum mesh, respectively. The measurements were made in a 250 cm^3^ flat corrosion cell (BioLogic, Seyssinet-Pariset, France), equipped with the openings that are necessary for the electrodes mounting. The working electrode was contacted by the corrosion medium via flat rubber O-ring (exposed surface area = 0.283 cm^2^) and the temperature was kept constant by a thermostat. A potentiostat-galvanostat (PARSTAT 4000, Princeton Applied Research, Ametek, Berwyn, PA, USA) that was controlled by the VersaStudio dedicated software v. 2.52.3 was used in the experiments. Prior to the investigations, the open-circuit potential (*E*_OC_) of the samples was stabilized for 5 h. The following measurements were made:

- electrochemical impedance spectroscopy (EIS) at *E*_OC_, the amplitude of 10 mV (RMS—root mean square), in the frequency range of 100,000–0.01 Hz and 10 points per decade of frequency. The experiment took approximately 1 h; and,
- potentiodynamic polarization (PDP) scan (0.167 mV/s) from −30 mV vs. *E*_OC_ to 2000 mV vs. SCE and a reverse scan to −200 mV vs. *E*_OC_. If the oxide breakdown was observed in the experiment the reverse scan was commenced after its detection.

Immediately after the EIS, the *E*_OC_ stabilization was adopted (approx. 30 min). The EIS results were fitted to equivalent electrical circuit (EEC) models to acquire values of parameters corresponding to different parts of the coatings or electrochemical interfacial processes. The fitting was done using a complex non-linear least square (CNLS) method by ZSimpWin v. 3.60 (Ametek, EChem software, Ann Arbor, MI, USA). Three to four parallel samples were used for each studied surface to ensure reproducibility.

## 3. Results and Discussion

### 3.1. Plasma Electrolytic Oxidation

Zirconium surface after the etching was visibly smoothed and acquired a degree of luster (Figure 1a), however, at the microscopic scale, the grained structure was revealed (Figure 1b). Such a long etching time (2 min) was necessary since shortening of this process resulted in the formation of dark residue composed of corrosion products of Zr and the remainder of the SiC particles after the grinding. 

As it was discerned from the 3-D roughness mapping (Figure 1c), the bare Zr surface was far from being flat, nonetheless, the grinding marks were completely removed, and the surface was highly reproducible.

**Figure 2:**
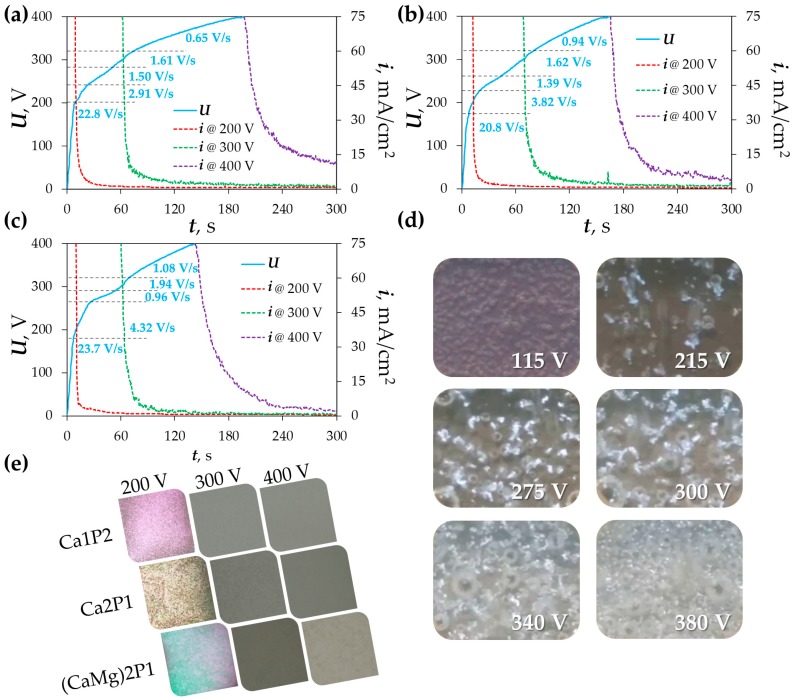
Voltage (*U*) and current density (*i*) transients recorded during the galvanostatic and potentiostatic steps of the PEO treatments in the Ca1P2 (**a**), Ca2P1 (**b**), and (CaMg)2P1 (**c**) solutions; macroscopic views of the zirconium surface at different points of the PEO process in the (CaMg)2P1 solution (**d**); and surface appearances after the treatment (**e**).

During the PEO, the anodization voltage (*U*) and the anodic current density (*i*) variation with time were recorded for all three electrolytes (Figure 2a–c). Five separate voltage-progression stages could be discerned from the plots. For the Ca1P2 solution, these regions were in the ranges: 0–200, 200–240, 240–280, 280–320, and 320–400 V (Figure 2a). In the case of the Ca2P1 solution, the regions were as follows: 0–180, 180–230, 230–260, 260–320, and 320–400 V (Figure 2b), while for the (CaMg)2P1 electrolyte the slopes changed in the ranges: 0–180, 180–265, 265–290, 290–320, and 320–400 V (Figure 2c). Each change in the slope of the *U* vs. *t* curve was associated with specific surface observations (Figure 2d). The stage I of the PEO process typically proceeds similarly to the conventional anodizing (Figure 2d; 115 V). This stage, when conducted galvanostatically, comprises the continuous thickening of the oxide film. It is manifested as color changes of the treated surface and sharp, linear voltage increase. 

The color changes were owed to the light interference on the uniformly growing oxide layer in the visible range [60], while the decrease in the interfacial conductivity (hence the voltage increase) was due to the dielectric oxide growth. During this stage, a relatively low rate of evolution of gaseous oxygen was also noted. From the results presented in Figure 2, it can be seen that the slope of the *U* vs. *t* curve varied slightly with the changes in the electrolyte composition. Generally, it was higher in the electrolytes having higher ionic strength. Similar observations were noted for the PEO of Ta in the comparable electrolytes [57]. However, it was found that the addition of magnesium acetate to the first solution led to the higher rates of voltage increase than those that were observed after the addition of calcium formate (14.1 and 16.0 V/s, respectively). Furthermore, in our previous report concerning the PEO of Nb, this effect was even more pronounced [56]. Therein, the use of the less concentrated solution containing Mg(CH_3_COO)_2_ resulted in a faster *U* increase than in the case of the more concentrated electrolyte that was containing the Ca salt. This behavior was explained by the reductive properties of formate ions, which slightly hindered the oxidation of the substrate. At 180–200 V, the zirconium substrates were observed to undergo dielectric breakdown and small bluish-white microdischarges (MD) were spotted moving rapidly across on the treated surfaces (Figure 2d; 215 V). It was the stage II of the process and it was relatively short-lived. After a few seconds, the slope of the voltage-time progression was significantly lowered, which was associated with a sharp increase in the oxygen evolution rate (stage III; Figure 2d, 275 V). In time, the MDs began to coalesce into larger clusters, which were brighter, moved much more slowly and grew larger in size (stage IV; Figure 2d; 300 V). The voltage-time slope increased again, suggesting that the higher plasma coverage increased the oxide sealing and/or the thickening rate. When the clusters were large enough to join one another and to form a net, the stage V of the process was discerned. It was observed as a gradual increase of the plasma surface coverage until the specimen was completely engulfed by the MDs (Figure 2d; 340 and 380 V). The similar voltage-time curves were observed by Matykina et al. [28] during DC PEO of titanium in the electrolyte that contained 5 g/dm^3^ of Na_3_PO_4_. They found that the oxide breakdown on Ti occurred at ca. 285 V, which is close to the results that are found herein. Furthermore, the observations of the plasma events on the treated surfaces were also similar. There is little data on the DC PEO *U* vs. *t* curves of zirconium or its alloys in the literature [32,38] apart from that belonging to the pulsed variant of the process [41,45,48,49]. The curve for the latter type of the treatment differs significantly in its shape and surface plasma events from the pure DC regime. For instance, the breakdown voltage at the frequency of 50 Hz is typically above 400 V.

In general, it was observed that the addition of Ca and Mg salts into the base electrolyte (Ca1P2) shortened the galvanostatic process step because stages II, IV, and V were observed to have higher *U* vs. *t* slopes than those that were observed in Figure 2a. When the potentiostatic step was reached the oxide layers begun to seal and stabilize under the constant voltage (sharp current density drop). The oscillations in the measured current were associated with the MDs that were still occurring until the end of the process. This effect was more pronounced at higher limiting anodization voltages because the sparks energy was also higher. The effect of the treatment in relation to the chosen electrolyte and *U*_L_ on the surface appearance of zirconium can be seen in Figure 2e. The samples PEO-ed at *U*_L_ = 200 V retained some degree of luster, nevertheless, they were observed to be dotted with darker regions. Interference colors were still evident, which meant that the oxide breakdown did not occur on the entire surface. After the treatment at 300 V or 400 V, the surfaces were uniformly coated with white or gray oxide coatings. Among these, the Ca2P1 300 and (CaMg)2P1 400 samples were the least homogeneous, with some dark or bright spots.

### 3.2. Surface Morphology and Elemental Composition

**Figure 3:**
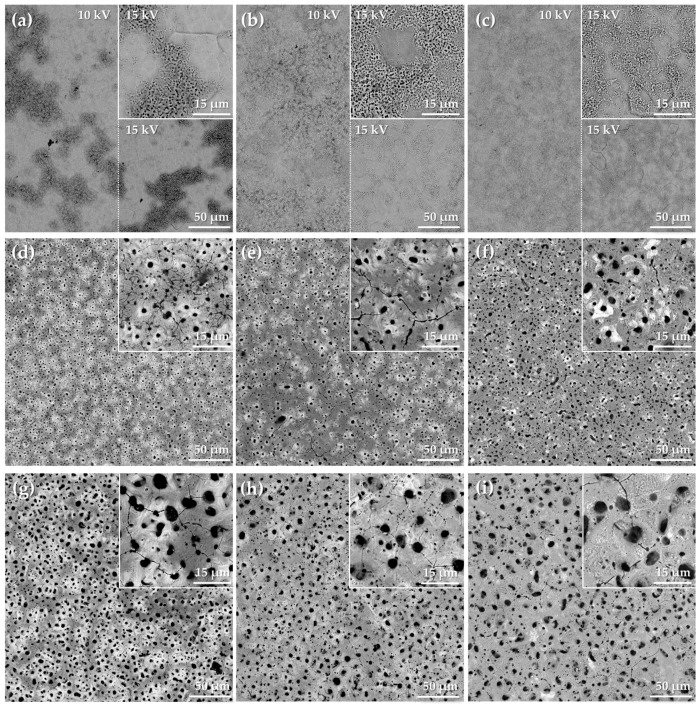
Surface morphologies of the PEO coatings formed on zirconium up to 200 (**a**–**c**), 300 (**d**–**f**) and 400 V (**g**–**i**) in the Ca1P2 (**a**,**d**,**i**), Ca2P1 (**b**,**e**,**h**), and (CaMg)2P1 (**c**,**f**,**i**) solutions. In the case of the samples that were treated up to 200 V half of the image is presented under accelerating voltage of 10 kV, while the other half was taken at 15 kV. The insets correspond to the larger magnifications of the same images.

The oxide coatings produced by PEO were observed under SEM and the results are shown in Figure 3. The accelerating voltage used for the viewing of the oxide coatings determines the imaging depth and the resolution of the image [61]. The surface morphology of the samples that were formed by PEO at *U*_L_ = 200 V (Figure 3a–c) was not homogeneous. Patches (or islands) of porous PEO oxide could be observed on the treated surfaces. The reason for such behavior is linked with the plasma regime that was observed around 200 V for all three electrolytes (Figure 2a–c). When the treatment voltage is high enough to induce the oxide breakdown the MDs appear [25,29]. However, as the *U*_L_ = 200 V was attained, the voltage was not allowed to grow higher and the current density dropped down. Consequently, the energy flux was too low to sustain the MDs, and the breakdown on the entire surface was not complete. The smallest porous oxide coverage was discerned for the Ca1P2 200 sample because the stage II of the treatment started at 200 V, and the MDs were quenched almost immediately after they appeared (Figure 2a). Interestingly, the oxide was found not to resemble the etched zirconium surface and the underlying substrate was only visible when the accelerating voltage was increased to 15 kV. The grained structure was the most prominent in the case of the Ca1P2 200 sample, which suggested that the oxide layer of this sample had the smallest thickness among the studied PEO films. The contrast in the SEM images signify the compositional changes, where the brighter spots correspond to the heavier elements, and conversely, dark spots are where the lighter elements are found [62]. The coatings produced at *U*_L_ = 300 V were found to cover the entire zirconium specimens (Figure 3d–f). However, small cracks were observed on their surfaces. The lighter regions of the coatings were zirconium-rich. This might be explained by the presence of so-called “B”-type microdischarge, leading to the partial melting of the underlying metal and its ejection through the plasma channel onto the oxidized surface, where it undergoes combined electrochemical and thermal oxidation. 

Conversely, the darker points may correspond to the evaporation of water in the peripheral region of the MDs, where the electrolyte constituents might crystallize and undergo thermal transformations into new compounds. It was found that the coatings, which were formed from the Ca1P2 solution (Figure 3d), were less homogeneous than their counterparts from the other electrolytes (Figure 3e,f). The similar relationship was encountered for the coatings produced at *U*_L_ = 400 V (Figure 3g–i). Furthermore, the pore size was mostly determined by the anodization voltage (hence, the intensity of sparking during the process), while their number depended more on the ionic strength of the electrolyte. The pores were formed by the generation of MDs and the detachment of oxygen bubbles from the growing oxide [25,28]. It is noteworthy that the cracks in the samples that were prepared at 400 V were partially melted and sealed, which was most likely due to the high temperature of the surface sparks.

**Figure 4:**
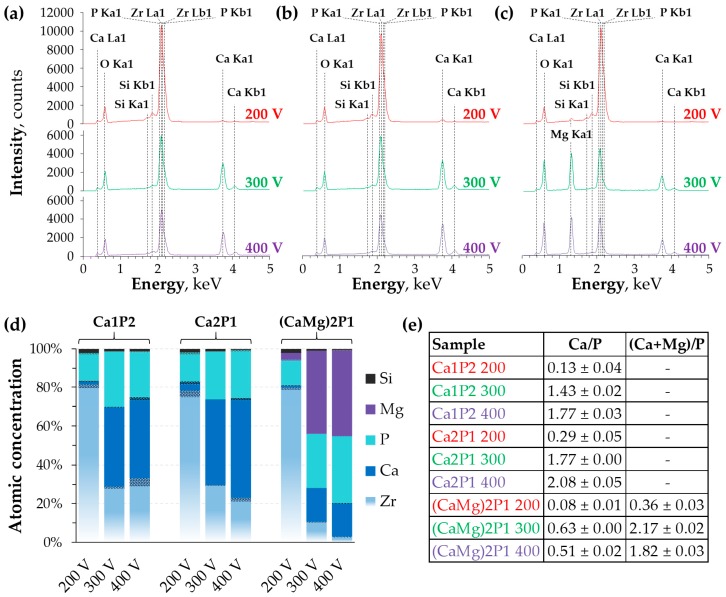
Energy-dispersive X-ray spectrometer (EDX) spectra obtained for the zirconium samples after PEO in the Ca1P2 (**a**), Ca2P1 (**b**), and (CaMg)2P1 (**c**) solutions. Relative atomic concentrations of the coatings’ surface (**d**) were calculated excluding oxygen signals. Dotted regions in (**d**) represent standard deviations (SD) of the composition determination. The EDX signals were used to obtain Ca/P and (Ca+Mg)/P atomic ratios of each PEO coating (**e**).

EDX analysis of the SEM images was performed to assess the surface elemental composition of the PEO coatings and the results are presented in Figure 4. It was found that the samples that were anodized at 200 V were enriched with the electrolyte components to only a small extent. 

This is due to relatively low charge passed through the surfaces, combined with the small energy of the MDs just after the breakdown voltage was reached (Figure 2a–c). The bar graph in Figure 4d shows the relative changes in the concentration of the surface elements in the oxide layers. Oxygen was not included in the calculations as its atomic weight is too small for this element to be quantifiable by EDX. From the data, it can be seen that phosphorus is much easier to incorporate into coatings than calcium and magnesium. Negatively charged hypophosphite ions are much more likely to incorporate into positively charged anode than the Ca^2+^ and Mg^2+^ ions. The degree of incorporation of the latter two was higher at higher voltages when the MDs were more energetic. Furthermore, less and less zirconium was present on the coatings’ surface as the voltage was increased. The Ca/P and (Ca+Mg)/P atomic ratios that are presented in Figure 4e indicate that the elemental composition of the coatings was closely related to that of the solutions. Magnesium was also found to be the dominating PEO electrolyte component introduced from the solution in the coatings that were prepared by Ha et al. [46], with the Ca/Mg atomic ratio in the coating being the same as the proportion of these elements in the bath. Nonetheless, the surface morphology of the films that were obtained in their work was different from that presented here. However, the presented results must be considered with care because of the difficulty of distinguishing between the EDX signals corresponding to zirconium and phosphorus, which complicates the quantification of these two elements [63].

**Figure 5:**
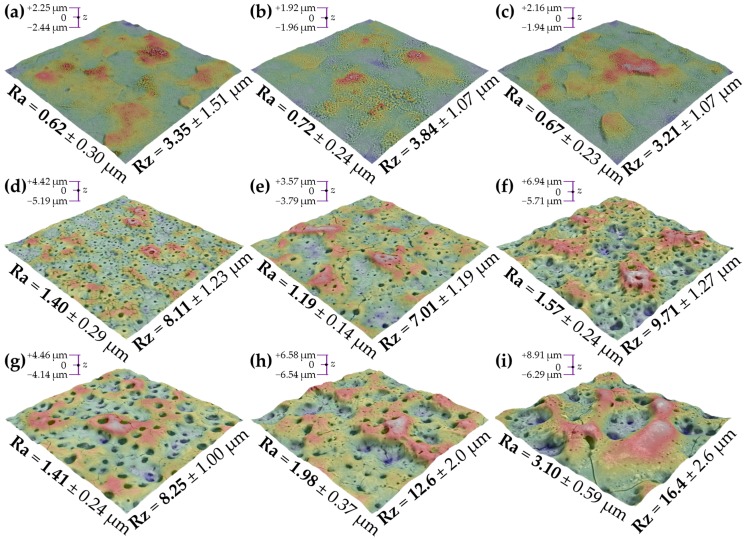
Scanning electron microscope (SEM) surface roughness mapping images of the PEO coatings formed on zirconium up to 200 (**a**–**c**), 300 (**d**–**f**), and 400 V (**g**–**i**) in the Ca1P2 (**a**,**d**,**i**), Ca2P1 (**b**,**e**,**h**), and (CaMg)2P1 (**c**,**f**,**i**) solutions. The images have dimensions of 90 µm × 90 µm, while the Ra and Rz values were calculated from the profiles taken from other images having the size of 270 µm × 270 µm.

Three-dimensional (3-D) roughness mapping of the oxide coatings was also realized by SEM (Figure 5). As it was determined earlier from the surface morphology investigations (Figure 3a–c), the samples after PEO at *U*_L_ = 200 V were smoothed slightly by the treatment (Figure 5a–c) and their surface relief was comparable to one another. 

At 300 V, the roughness increased with the increasing ionic strength of the solution (Figure 5d–f). While in the case of the samples after PEO at 400 V, it was found that the presence of the Ca and Mg salts in the electrolytes was more effective at increasing the surface roughness of zirconium than their concentration. It was found that at this *U*_L_, in addition to the surface pores, oblong strut-like bulges contributed to the roughness of the PEO coatings that were formed from the Ca2P1 and (CaMg)2P1 solutions, which were also observed in the case of Ta and Nb specimens [56,57].

### 3.3. Coatings’ Thickness and Structure

**Figure 6:**
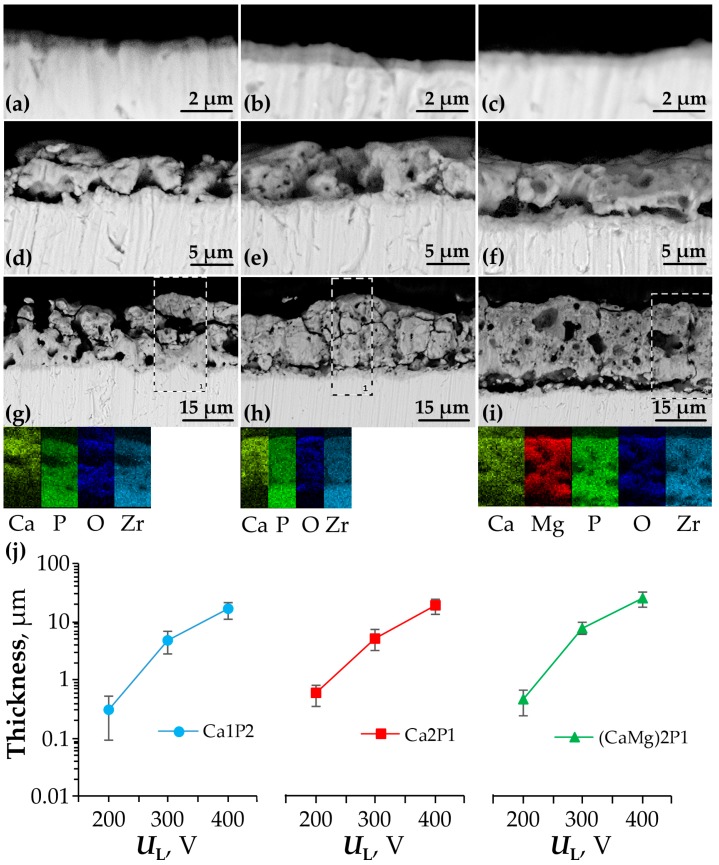
SEM cross-sections of the PEO coatings formed on zirconium up to 200 (**a**–**c**), 300 (**d**–**f**), and 400 V (**g**–**i**) in the Ca1P2 (**a**,**d**,**i**), Ca2P1 (**b**,**e**,**h**), and (CaMg)2P1 (**c**,**f**,**i**) solutions. Rectangles in the images of the coatings produced at 400 V represent the regions of EDX elemental mapping, results of which are presented directly below the corresponding images. The ranges of the measured thickness (**j**) were calculated as mean ± SD for each coating.

Cross-sectional views of the PEO coatings served to investigate their structure and thickness (Figure 6). 

The oxide coatings on Zr specimens that were treated by PEO at *U*_L_ = 200 V appeared to have the similar structure (Figure 6a–c), resembling the amorphous thin oxide films that were typically encountered for conventionally anodized Al and Ti alloys [64,65]. It was determined that the thickness was larger in some points of the coatings than in the others, which is probably because of the porous oxide islands, which were seen in the planar SEM views in Figure 3a–c. The mean thickness of the films formed at 200 V was 302, 584, and 454 nm for the Ca1P2, Ca2P1, and (CaMg)2P1 electrolytes, respectively. The nonuniformity in their thickness can be seen from relatively large ranges in Figure 6j, depicted as error bars. The coatings obtained at 300 and 400 V in the Ca1P2 and Ca2P1 solutions were of comparable thickness (mean 4.89, 16.3 µm, and 5.16, 18.7 µm, respectively; Figure 6d,e,g,h), while those that were formed in the (CaMg)2P1 electrolyte were significantly thicker (mean 7.92 and 25.6 µm; Figure 6f,i). Nevertheless, the structure of the PEO films was comparable to one another in the case of the Ca2P1 and (CaMg)2P1 solutions. The coatings prepared in the Ca1P2 solution had the largest error bars (Figure 6j) among the studied samples, which indicate the high nonuniformity of their thickness (although, it was decreasing with the increasing voltage). In terms of their structure, the coatings were characterized by a bilayer character. The outermost oxide layer was highly porous (the porosity increased with the voltage) and thick, while the barrier layer resided at the oxide-metal interface. Such an arrangement is typical for the PEO oxide films [36,42,45,50,51,66]. The EDX mapping in Figure 6g–i indicate that mostly all elements are uniformly spread across the coatings’ thickness. The only exceptions are Mg and Ca, which were slightly more concentrated in the outermost parts of the coatings. It is in accordance with the finding that the enrichment of the zirconium surface with the positive ions was higher at higher voltages (Figure 4d). Such consolidation of some of the electrolyte components near the electrolyte-coating interface was also reported by others [34,43].

### 3.4. Electrochemical Impedance Spectroscopy

**Figure 7:**
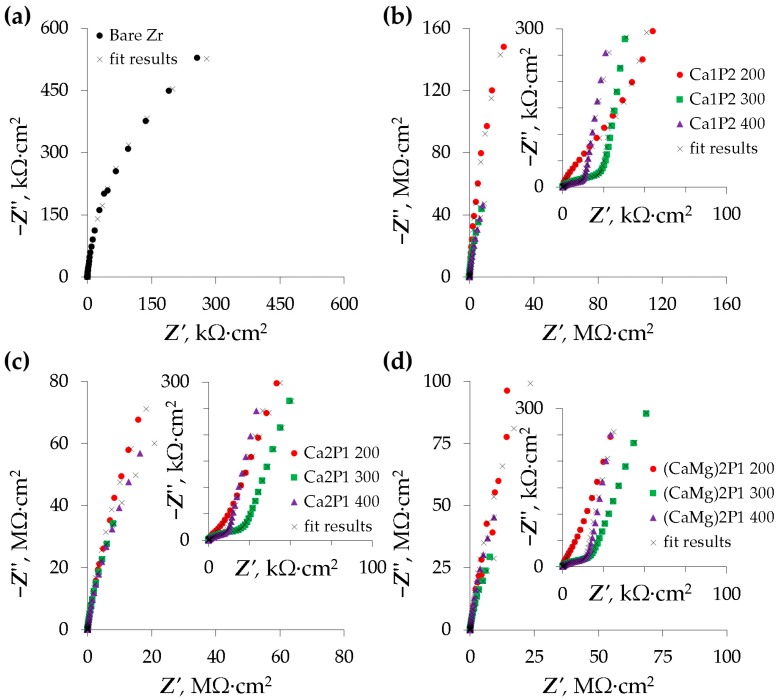
Complex plane Nyquist plots of impedance spectra belonging to the bare zirconium surface (**a**) and the PEO coatings formed in the Ca1P2 (**b**), Ca2P1 (**c**), as well as (CaMg)2P1 (**d**) solutions.

**Figure 8:**
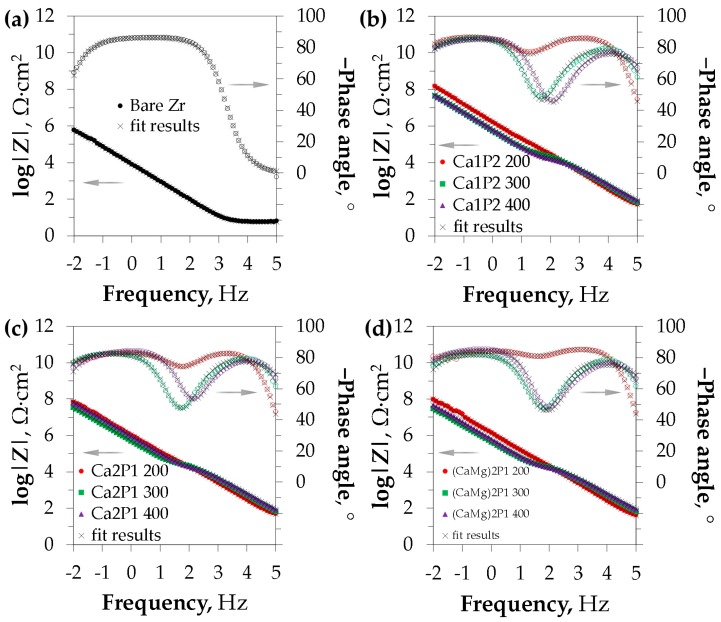
Bode modulus (**|*Z*|**) and phase angle plots belonging to the bare zirconium surface (**a**) and the PEO coatings formed in the Ca1P2 (**b**), Ca2P1 (**c**), as well as (CaMg)2P1 (**d**) solutions. Arrows indicate the data sets that correspond to the specific axes.

Corrosion resistance investigations comprised the electrochemical impedance spectroscopy studies, as well as potentiodynamic polarization tests. The first of the methods is a non-destructive technique of probing the electrode-electrolyte interface and provides the means of determining the contribution of a variety of factors (such as the presence of oxide film, its resistance, and capacitance, as well as diffusional limitations) to the total impedance of the corroding system [67]. The EIS results are presented in the form of Nyquist complex plane plots (Figure 7) as well as Bode impedance modulus/phase angle plots (Figure 8).

From the spectra in Figure 7, it can be inferred that the studied surfaces exhibited one or two capacitive loops, which is typical for the corroding metal systems. The bare zirconium sample was characterized by a single loop that was clearly visible in Figure 7a. The spectra belonging to the Zr coated by the PEO layers were slightly more complicated. For the coatings formed in the Ca1P2 electrolyte (Figure 7b), two time constants were discerned from two distinct capacitive loops. It is noteworthy that the size of the high-frequency loops (inset in Figure 7b) decreased with the limiting anodization voltage. Similar features and trends were found in the spectra belonging to the coatings that were obtained from the other solutions as well (Figure 7c,d).

The spectra of the PEO coatings were shown to exhibit higher impedance than that of the bare zirconium surface in the entire frequency range, as it can be deduced from Figure 8. This was the evidence of their good anti-corrosion performance. Furthermore, the corrosion resistance of the thin oxide films (obtained at *U*_L_ = 200 V) was superior to that of the thick coatings that were obtained at 300 and 400 V. The difference in |*Z*| between the latter two was insignificant in the case of the Ca1P2 electrolyte (Figure 8b). However, for the Ca2P1 and (CaMg)2P1 solutions, it was found that the treatment at 400 V led to the higher impedance of the system as compared to that conducted at 300 V (Figure 8c,d). The similar shapes of the spectra were also obtained for Ta and Nb in the similar solutions systems [56,57] and for the titanium substrates with ZrO_2_-containing PEO coatings, as determined by Babaei et al. [68]. Nonetheless, the overall impedance of the coatings studied in this research was at least one order of magnitude higher compared to the cited reports.

**Figure 9:**
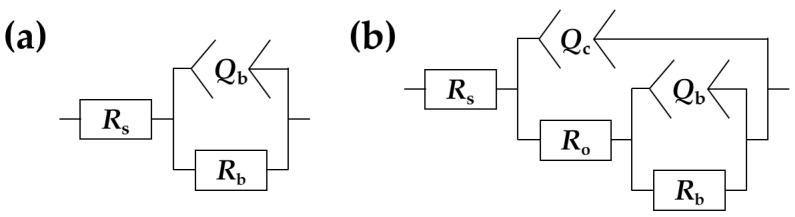
Equivalent electrical circuits (EECs) used for analysis of the electrochemical impedance spectroscopy (EIS) results corresponding to the bare zirconium surface (**a**) and the PEO coatings (**b**).

Data obtained from the EIS were fitted to two EECs that are shown in Figure 9. The first circuit (*R*(*QR*)) corresponds to the bare zirconium surface immersed in Ringer’s solution [69,70], while the second (*R*(*Q*[*R*(*QR*)])) models the corrosion behavior of the PEO coatings. *R*_s_ is the uncompensated ohmic resistance of the electrolyte solution and the wiring from the measuring device. This parameter is not crucial from the point of view of corrosion resistance determination, nevertheless, its presence is required for the fitting of the experimental data. Parallel connection between *R*_b_ and *Q*_b_ models the electrochemical interface between the electrolyte and the metal surface, where *R*_b_ is the resistance that is associated with the passive (or barrier) oxide layer on the zirconium substrate, while *Q*_b_ is the element approximating its capacitance. The additional *RQ* pair in the circuit corresponding to the PEO coatings was included in the model to account for the second time constant of the system, which is typically associated with the porous oxide layer on top of the barrier sublayer [71]. *R*_o_ is the resistance of electrolyte in the pores of the outer oxide layer and *Q*_c_ is modeling the capacitive behavior of the entire coating. In the case of the samples that exhibited the inhomogeneous porous microstructure of the coatings (at 200 V), it is expected that the corresponding *R*_o_ will be much higher since the measurement is averaged over the entire interface. The capacitance of the electrochemical systems (especially those under un-steady corrosion conditions) is rarely identical to that of the ideal capacitor. Therefore, it was required to take the non-ideality into consideration by replacing the ideal capacitors by the so-called constant phase elements (CPE). This is a general circuit element, the impedance of which is governed by the following expression [72]:

(3) $$Z_{\mathrm{CPE}}=1/Q{\left(j\omega \right)}^{n}\;,$$

where *j* is the imaginary unity, *ω* represents the angular frequency (*ω* = 2*πf*; where *f*—frequency), while *Q* and *n* are the CPE parameters. *Q* attains different meanings at the specific values of *n*. When *n* = 1, *Q* is identical to the capacitance of an ideal capacitor (*C*). If *n* is zero, then the inverse of *Q* is the same as the resistance (no imaginary part). In the event when *n* = −1, then *Q*^−1^ = *L*, where *L* corresponds to the ideal inductance. In the whole range between −1 and 1, *Q* is approximating the behavior of one circuit element or the other.

The fitting results are shown in Table 1. The quality of the fit can be assessed by reviewing the positions of the experimental points (geometric symbols) and the calculated points (black crosses) in Figure 7 and Figure 8. In addition, the values of *χ*^2^ are summarized in Table 1. In general, the values of chi-squared were in the order of 1 × 10^−3^. It is evident from the results that the main factor in determining the corrosion resistance of the PEO-treated samples is the barrier layer resistance (*R*_b_), which is in accordance with the prevailing literature on the topic [73,74,75]. There seems to be the tendency of increasing of the barrier layer resistance in with the voltage in the order: 300, 400, and 200 V. This relationship can be compared with the *U* vs. *t* curves from Figure 2. The samples Ca1P2 200, Ca2P1 200, and (CaMg)2P1 200 were all treated up to a voltage just after the attaining of the breakdown potential when the MDs were barely visible on the surface. From the SEM images of the samples, it can be seen that the breakdown did not occur uniformly on the entire surface of the samples (Figure 3a–c). Therefore, the protective properties of the barrier layer were mostly retained at this point. Additionally, the pores on their surfaces were relatively small, thereby limiting the corrosion medium ingress. The coatings that were obtained at *U*_L_ = 300 V were all in the stage IV of the oxidation (Figure 2), where the sparks were concentrated in the weakest spots of the oxide, growing in size, and intensifying with time. These led to the loss of the tightness of the barrier oxide and significantly increased the interfacial area, which both result in a decrease of *R*_b_ (Table 1). As the process transitioned from stage IV to stage V, the treated surface was gradually covered with fine and intensive sparking. In consequence, the coatings that were obtained at 400 V were sealed to a greater extent than those that were formed at 300 V, however, their resistance was not as large as that of the barrier layer films on zirconium that was prepared at 200 V. Although the *R*_o_ of the PEO coatings does not influence the overall resistance in a significant way, it is possible to infer from the results the relative porosity of the porous sublayer. It was found that the pore resistance of the PEO coatings was decreased at higher voltages (Table 1, Figure 7 insets), leading to the statement that their internal porosity increased with the voltage. The highest *R*_o_ was found for the Ca1P2 200 sample, while the lowest belonged to the Ca1P2 400 sample. However, the differences in this parameter for the coatings that were formed at 400 V in each electrolyte were within experimental error (Table 1). 

The value of *n* of a CPE determines how its behavior deviates from the ideal circuit element (e.g., capacitor). This deviation stems from the chemical inhomogeneity and unevenness of the interface in question. The values of *n*_c_ of the PEO films departed from the unity to a greater extent with the increasing anodization voltage, which is in agreement with the microroughness results (Figure 5). Furthermore, the effective capacitance of a CPE (*C*_c_) can be estimated from *Q* and *n* by means of the following relationship [72,76]:

(4) $$C_{c}=\sqrt[{n}]{QR}/R\;,$$

where *Q* and *R* must be electrically connected in parallel (only for *n*→1). It follows from the definition of the capacitance that it is proportionally related to the surface area (*A*) of the electrochemical interface, and it is inversely proportional to the thickness of the dielectric (*d*):

(5) $$C=\frac{\varepsilon \varepsilon_{0}A}{d}\;,$$

where *ε* is the dielectric constant and *ε*_0_ is the permittivity of the free space. Therefore, the capacitance of the PEO coatings should decrease with the increasing thickness. Nevertheless, during the experiments, the pores of the coatings were filled with Ringer’s solution, which had much larger dielectric constant than the oxide films. As a result, the samples after the PEO at 300 and 400 V had higher capacitances than those that were treated at 200 V (Table 1), although the thicknesses of the former two were much higher. Since the thickness and the roughness (i.e., the interfacial surface area) of the coatings that are presented here was governed mainly by the anodization voltage it is possible to determine the degree of the oxide films penetration by Ringer’s solution by comparing the values of *C*_ef_ obtained at 200, 300, or 400 V. Consequently, it was found that among all of the oxide coatings, the films that were formed in the electrolyte containing Mg^2+^ ions were penetrated by the electrolyte to the greatest extent. The similar relationships were found for Ta and Nb in our previous studies [56,57].

### 3.5. Polarization Experiments

**Figure 10:**
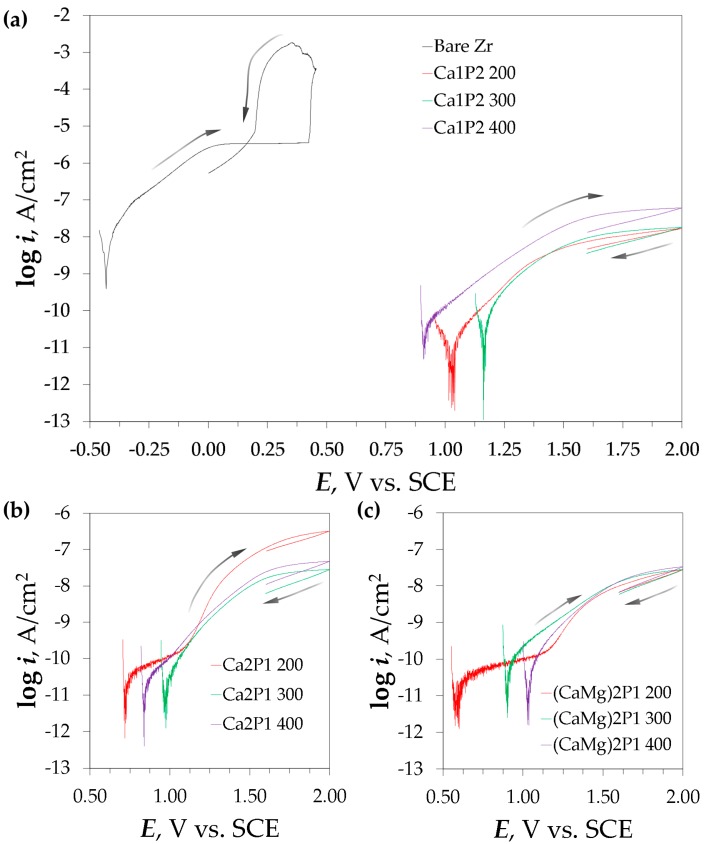
Potentiodynamic polarization curves recorded for the bare zirconium surface and the PEO coatings formed in Ca1P2 (**a**), Ca2P1 (**b**), as well as (CaMg)2P1 (**c**) solutions. Arrows indicate the direction of the potential scans.

Immediately after the EIS studies, a short period of potential stabilization (approx. 30 min) was adopted. Then, the coatings were subjected to potentiodynamic polarization scans, the results of which can be seen in Figure 10. The PDP curve for the bare zirconium surface had a few characteristic features (Figure 10a). After the transition of the curve through the corrosion potential (−446 ± 28 mV vs. SCE), the current was observed to obey the linear Tafel kinetics in the potential range −300–0 mV vs. SCE, which is associated with an active dissolution region of the curve. Therefore, it is found that the metal was under active dissolution state in the experimental conditions described here. At the potential of +100 mV vs. SCE, the passivation plateau was observed, and it continued up to the breakdown or pitting potential (*E*_break_ = 412 ± 39 mV vs. SCE). The oxide breakdown initiation resulted in a sharp increase in the measured current and this was the point at which the polarization direction was reversed. In the backward scan, the current was observed to rise and then it dropped sharply at the so-called pit protection potential (*E*_prot_ = 173 ± 8 mV vs. SCE), which corresponds to the corrosion pits repassivation [77,78]. Chen et al. [13] studied the effect of chloride concentration on some of the corrosion parameters of zirconium and Zr-2.5Nb alloy. The potential ranges (*E*_cor_, *E*_break_, *E*_prot_) that were determined in their studies agree with those obtained here. In addition, the breakdown potential that was obtained for Zr in SBF (Simulated Body Fluid) found in the literature lies between 340 and 450 mV vs. SCE [47,48]. Furthermore, it has to be taken into account that in the case of immersion of the metal in the real physiological conditions, the medium could exert more oxidizing effect on the surface, which would shift the corrosion potential of zirconium into the passive region, which would explain its good biocompatibility [7,9,10,11,12]. The PDP curves belonging to the PEO-treated zirconium specimens had their corrosion potentials shifted significantly to the anodic direction, and the measured currents were at least two orders of magnitude lower (Figure 10). Furthermore, there was no instance of breakdown observed in the case of the coated samples. The oxidation waves observed for the curves at ca. 1 V vs. SCE were associated with the gaseous oxygen evolution, which was also reported for Ti-13Nb-13Zr alloy investigated in Hanks’, Eagle’s, and Ringer’s physiological solutions [79,80], as well as for Ti, Ta, Nb, and Ti-20Nb-10Zr-5Ta alloy that was measured in Hanks’ medium [81]. The currents measured in the reverse scans were always lower than those in the forward scans, which proves that the polarization led to the sealing of the investigated interfaces rather than their breakdown. 

Some corrosion parameters of the zirconium specimens that were measured in the Ringer’s solution are presented in Table 2. *R*_p_ is the polarization resistance, which is defined as the slope of the *i* vs. *E* relationship in the limit of ±10 mV vs. *E*_cor_. Apart from the polarization curves, the *R*_p_ can also be obtained from the EIS data by summing up the real components’ contributions from the EECs from Figure 9. Therefore, *R*_p,EIS_ for the bare zirconium sample can be defined as *R*_s_ + *R*_b_, whereas for the PEO-treated zirconium samples, this parameter can be calculated as *R*_s_ + *R*_o_ + *R*_b_ [73]. The passivation current density (*i*_pas_) was defined here as the current density just before the onset of a breakdown in the case of the bare Zr samples and for the PEO-coated samples, it was taken as the current density at the peak potential of the polarization curves (i.e., 2 V vs. SCE).

There was a significant spread in the values of *E*_cor_ for the Zr samples after PEO, none of which was differing from one another in a statistically significant way (Table 2). Additionally, it was found that the polarization resistance that was determined via DC methods was consistently higher than that obtained from the EIS results, however, the trends in the data are similar. The tightness of the oxide coatings was assessed by the value of *i*_pas_ [71]. Among the coatings that were obtained at *U*_L_ = 200 V, the one that was formed in the Ca2P1 electrolyte exhibited the worst corrosion resistance and its *i*_pas_ was one order of magnitude higher with respect to the other two. This effect was probably due to the reductive properties of formate ions that were present in the solution. Although, this coating was found to be the thickest among those obtained at 200 V (Figure 6a–c,j), it was also found to be the most porous, since the *R*_o_ corresponding to this surface was the smallest (Figure 7, Table 1). The differences between the coatings formed at higher voltages were less significant. The tightest coatings were obtained from the Ca1P2 solution at 200 and 300 V. However, the process voltage had the least effect in the case of the (CaMg)2P1 electrolyte, where even for the coating that was treated at 400 V, the value of *i*_pas_ remained relatively low. These findings can be compared with the surface morphology investigations (Figure 3), where it was found that the addition of the Ca and Mg salts to Ca(H_2_PO_2_)_2_ increased the intensity of the sparks between 300 and 400 V to such a point that the surface cracks were partially sealed (Figure 3g–i). The sealing was better for the (CaMg)2P1 solution, suggesting that the higher concentration of the electrolyte supported this effect.

## 4. Conclusions

The work that was described here was concerned with the direct current plasma electrolytic oxidation of commercially pure zirconium performed in a galvanostatic/potentiostatic manner. The treatment was carried out in three solutions that contained Ca(H_2_PO_2_)_2_, Ca(HCOO)_2_, and Mg(CH_3_COO)_2_ up to 200, 300, or 400 V. Five stages of anodic oxidation were identified during the treatment and their effect on the corrosion resistance of the resulting PEO-coated samples was determined. It was found that the PEO improved the corrosion performance of Zr (pitting corrosion resistance in particular), irrespectively of the applied process conditions. The thickness and the roughness of the coatings were increasing with the limiting anodization voltage and the electrolyte composition had only a little effect on these surface characteristics. A significant incorporation of the positively charged ions into PEO layers was observed at 300 and 400 V. The oxide films had two sublayers: the innermost barrier layer and the outer porous layer. The properties of the obtained coatings were mostly characterized by the plasma regime that was observed at the end of the treatment. The best corrosion resistance was obtained for the Ca1P2 200 sample; however, its surface morphology was altered only slightly when compared with the untreated Zr surface. Among the fully developed porous PEO oxide coatings, the best corrosion resistance was discerned for the Ca1P2 300, Ca2P1 300, and (CaMg)2P1 400 samples, of which the latter two had the chemical composition the most favorable for the bioactive surfaces (Ca/P or (Ca+Mg)/P ratios that were close to that of hydroxyapatite). Additionally, the PEO oxide films that were prepared in the (CaMg)2P1 solution had the largest capacitances among all of the studied coatings, which was linked with the easier penetration of Ringer’s solution into the pores of the Mg-rich oxide layers at all the studied voltages.

## Figures and Tables

**Table 1:** Circuit parameters that were obtained from the fitting of the EIS data to the EECs from Figure 9.

Sample	*R*_s_ Ω·cm^2^	*Q*_c_ × 10^8^ s*^n^*/(Ω·cm^2^)	*n*_c_	*C*_c_ × 10^8^ F/cm^2^	*R*_o_ × 10^−4^ Ω·cm^2^	*Q*_b_×10^8^ s*^n^*/(Ω·cm^2^)	*n*_b_	*R*_b_ × 10^−8^ Ω·cm^2^	*χ*^2^ × 10^4^
Bare Zr	5.83 ± 0.13	-	-	-	-	2111 ± 45	0.96 ± 0.00	0.0135 ± 0.0020	<3.72
Ca1P2 200	35.4 ± 9.4	6.59 ± 0.04	0.97 ± 0.00	7.79 ± 0.18	34.6 ± 16.3	3.63 ± 1.83	0.97 ± 0.03	19.7 ± 5.5	<9.92
Ca1P2 300	19.6± 4.0	10.1 ± 0.3	0.89 ± 0.00	15.9 ± 1.4	3.99 ± 0.46	21.2 ± 3.8	0.99 ± 0.01	4.64 ± 1.61	<16.9
Ca1P2 400	15.0 ± 1.2	9.83 ± 0.69	0.88 ± 0.01	16.9 ± 1.6	2.06 ± 0.32	19.0 ± 4.7	0.99 ± 0.00	4.96 ± 1.48	<9.89
Ca2P1 200	31.4 ± 6.0	10.3 ± 1.8	0.94 ± 0.01	13.3 ± 3.1	7.05 ± 1.92	7.69 ± 3.19	0.89 ± 0.03	6.14 ± 1.76	<9.88
Ca2P1 300	20.1 ± 2.9	9.47 ± 1.02	0.90 ± 0.01	13.7 ± 2.0	3.46 ± 0.19	27.2 ± 5.3	0.94 ± 0.05	3.12 ± 0.41	<11.2
Ca2P1 400	16.2 ± 2.3	8.55 ± 0.13	0.89 ± 0.00	12.6 ± 0.3	2.47 ± 0.10	11.3 ± 1.3	0.97 ± 0.01	2.87 ± 0.50	<11.2
(CaMg)2P1 200	28.0± 1.1	8.99 ± 0.87	0.96± 0.01	10.6 ± 1.5	17.0 ± 9.8	3.76 ± 0.81	0.88 ± 0.06	8.71 ± 3.20	<59.2
(CaMg)2P1 300	18.4 ± 1.5	10.8 ± 0.4	0.89 ± 0.00	15.4 ± 0.8	2.64 ± 0.17	30.7 ± 5.9	0.93 ± 0.03	1.71 ± 0.34	<13.1
(CaMg)2P1 400	14.5 ± 2.2	11.2 ± 0.9	0.87 ± 0.01	20.8 ± 2.3	2.50 ± 0.39	17.7 ± 3.4	0.99 ± 0.01	4.73 ± 0.81	<13.6

**Table 2:** Corrosion parameters of zirconium before and after the PEO obtained from the potentiodynamic polarization (PDP) and EIS studies in Ringer’s solution.

Sample	*E*_cor_ mV vs. SCE	*R*_p_ GΩ·cm^2^	*R*_p,EIS_ GΩ·cm^2^	*E*_break_ mV vs. SCE	*E*_prot_ mV vs. SCE	*i*_pas_ nA/cm^2^
Bare Zr	−446 ± 28	0.00250 ± 0.00018	0.00135 ± 0.00020	412 ± 39	173 ± 8	3609 ± 100
Ca1P2 200	996 ± 28	2.92 ± 1.05	1.98 ± 0.55	-	-	13.8 ± 4.4
Ca1P2 300	1042 ± 117	0.490 ± 0.210	0.464 ± 0.161	-	-	16.1 ± 2.3
Ca1P2 400	950 ± 52	0.673 ± 0.073	0.496 ± 0.148	-	-	65.2 ± 12.6
Ca2P1 200	856 ± 163	0.714 ± 0.288	0.614 ± 0.176	-	-	440 ± 173
Ca2P1 300	941 ± 132	0.734 ± 0.231	0.312 ± 0.041	-	-	28.0 ± 3.9
Ca2P1 400	889 ± 100	0.610 ± 0.075	0.287 ± 0.050	-	-	59.0 ± 8.2
(CaMg)2P1 200	644 ± 70	1.40 ± 0.47	0.871 ± 0.320	-	-	33.3 ± 9.3
(CaMg)2P1 300	758 ± 199	0.374 ± 0.055	0.171 ± 0.034	-	-	31.9 ± 3.4
(CaMg)2P1 400	886 ± 228	0.617 ± 0.167	0.473 ± 0.081	-	-	40.3 ± 6.7
